# Total morphosynthesis of biomimetic prismatic-type CaCO_3_ thin films

**DOI:** 10.1038/s41467-017-01719-6

**Published:** 2017-11-09

**Authors:** Chuanlian Xiao, Ming Li, Bingjun Wang, Ming-Feng Liu, Changyu Shao, Haihua Pan, Yong Lu, Bin-Bin Xu, Siwei Li, Da Zhan, Yuan Jiang, Ruikang Tang, Xiang Yang Liu, Helmut Cölfen

**Affiliations:** 1 College of Materials, Research Institute for Soft Matter and Biomimetics, Xiamen University, Xiamen, 361005 China; 2 Qiushi Academy for Advanced Studies, Zhejiang University, Hangzhou, 310027 China; 3 Fujian Key Laboratory of Materials Genome, Xiamen University, Xiamen, 361005 China; 4 College of Chemistry & Chemical Engineering, Xiamen University, Xiamen, 361005 China; 5 Key Laboratory of High Performance Ceramic Fibers, Ministry of Education, Xiamen University, Xiamen, 361005 China; 6 Fujian Provincial Key Laboratory for Soft Functional Materials Research, Xiamen University, Xiamen, 361005 China; 7 State Key Laboratory of Marine Environmental Science, Xiamen University, Xiamen, 361005 China; 8 Department of Physics, Faculty of Science, National University of Singapore, 117542 Singapore, Singapore; 9 Physical Chemistry, University of Konstanz, Konstanz, 78457 Germany

## Abstract

Biomimetic mineralization can lead to advanced crystalline composites with common chemicals under ambient conditions. An exceptional example is biomimetic nacre with its superior fracture toughness. The synthesis of the prismatic layer with stiffness and wear resistance nonetheless remains an elusive goal. Herein, we apply a biomimetic mineralization method to grow prismatic-type CaCO_3_ thin films, mimicking their biogenic counterparts found in mollusk shells with a three-step pathway: coating a polymer substrate, deposition of a granular transition layer, and mineralization of a prismatic overlayer. The synthetic prismatic overlayers exhibit structural similarity and comparable hardness and Young’s modulus to their biogenic counterparts. Furthermore, employment of a biomacromolecular soluble additive, silk fibroin, in fabrication of the prismatic thin films leads to micro-/nano-textures with enhanced toughness and emerging under-water superoleophobicity. This study highlights the crucial role of the granular transition layer in promoting competition growth of the prismatic layer.

## Abstract (web summary)

The exterior layers of mollusk shells are prismatic in nature, endowing them with stiffness and wear resistance. Inspired by these biominerals, here, Jiang and colleagues grow structurally similar prismatic-type CaCO_3_ thin films with comparable stiffness and hardness.

## Introduction

The remarkable functionality of biominerals can be convincingly attributed to their complex and usually hierarchical architectures across many length scales^1^. Replication of representative biogenic structural forms by using biomimetic mineralization tools is therefore one of the most important thrusts in materials science^2,3^. Among various types of biogenic forms of crystalline biominerals, the pearly, nacreous layers and the chalk-like, prismatic ones commonly found in mollusk shells are very attractive targets for studies of biomimetic mineralization^4^. The reason is that the superior fracture toughness of nacre makes it one of the most studied biominerals and meanwhile, the layered design principle is inspiration for numerous scientists. Large-scale assembly methods have been used in the fabrication of free-standing, lamellar-type nanoplatelet-polymer hybrid thin films, which even possess superior mechanical properties to those of the biogenic nacreous layer^5–9^. However, only considering the toughness of the nacreous layer overlooks that mollusk shells exhibit stiffness and wear resistance due to the presence of an exterior prismatic layer. Such a type of prismatic-nacreous heterogeneous architecture is superior against non-uniform stress distributions. Inspired by the concept of spatial structural heterogeneity^10^, Erb et al.^8^ designed a bi-layered synthetic composite with an external layer with out-of-plane oriented platelets and an internal layer with in-plane oriented platelets. This composite displays the combination of hardness/wear resistance and strength/toughness by using the same basic building blocks—nanoplatelets.

While synthetic attempts remain limited in mimicking key structural features of the lamellar type^11–13^, production of biomimetic nacre nevertheless has been achieved very recently^14^. To date, no prismatic layer as a second key feature of mollusk shell architectures has been replicated in a reliable synthetic/assembly approach to CaCO_3_ thin films, which impedes the fabrication of biomimetic thin films with structural functions. Consequently, design of biomimetic mesostructural architectures with spatial prismatic-nacreous heterogeneity found in mollusk shells remains an elusive goal. Inspiringly, a careful scan at the structural spatial heterogeneity found in numerous biominerals provides crucial information, paving the way to the access of controllable synthetic approaches to biomimetic products^10^. For instance, Addadi and coworkers proposed that in larval shells of the marine bivalves, a granular layer seeded the mineralized columnar one^15^. Such a complex structural integration cancels out the crystallographic difference between the polymeric and mineralized components and hence, results in the significant decrease in interfacial energies in biogenic thin films with spatial structural heterogeneity. The presence of a transition layer was also verified between the adjacent prismatic and nacreous layers in mollusk shells^16,17^. Therefore, the presence of the granular transition layer is likely crucial for mimicking the key structural features of the biominerals and generating the continuous, highly-regulated overlayer. This assumption is strongly supported by the indispensable roles of the granular layer in the classical vapor deposition of columnar thin films^18,19^ and the emerging sol-gel approach to the prismatic- and lamellar-type thin films^20^. Despite the presence of intensively studied columnar ceramic thin films^18–20^, there lacks a rationally-designed, synthetic approach to achieving oriented mesostructural architectures in biomimetic mineralization studies. This can be convincingly attributed to the essential enrollment of multiple polymeric constituents, which function synergistically in a dynamic mineralization process. For instance, glycoproteins, chitin, and silk-like proteins in mollusk shells function as soluble additives, insoluble matrices, and gel-like media, respectively, in producing the nacreous-type mesostructural architecture^21^. Therefore, the main question in biomimetic mineralization lies in how to design a granular transition layer composed of both mineralized and polymeric constituents on an insoluble polymer matrix^22,23^ and whether it can facilitate the epitaxial growth of highly-oriented, hierarchical architectures continuous across the macroscopic distance. The success will in turn enrich our mechanistic understanding of biomineralization and meanwhile, provide a reliable synthetic approach to biomimetic hybrids with distinct mesostructures and remarkable structural functions at ambient conditions.

Herein, we report a total synthetic approach to the continuous, highly-oriented, prismatic-type CaCO_3_ thin films, taking advantage of the overgrowth on the granular transition layer following the competition growth model.

## Results

### Process overview

**Fig. 1:**
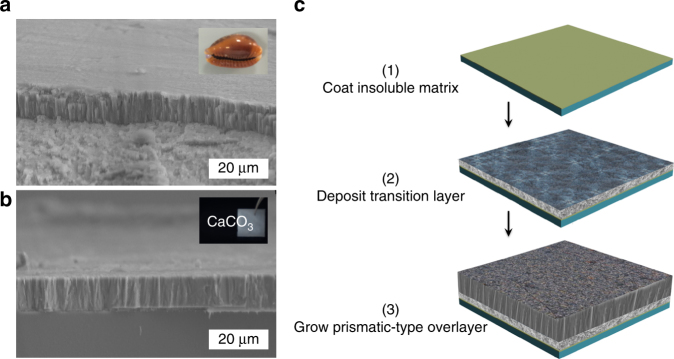
Synthetic approach to prismatic-type thin films and their structural similarity to biogenic counterparts. **a** Cross-section SEM image of the exterior prismatic-type layer in *Monetaria annulus* (the inserted photo is 1.0 cm in length). **b** Synthetic CaCO_3_ thin films fabricated in the current study. The CaCO_3_ logo is visible under a macroscopic uniform and translucent CaCO_3_ thin film deposited on the cover glass 1.2 cm in length. **c** Schematic outline of the three-step procedure used to grow the synthetic, prismatic-type thin films

The synthetic minerals show remarkable structural similarity to an exemplary biogenic counterpart—the prismatic-type, columnar layer of *Monetaria annulus* (Fig. 1a, b). The fabrication procedure was performed as follows (Fig. 1c). A poly(vinyl alcohol) (PVA) thin film was first deposited via a solution casting approach, followed by an annealing procedure (see Methods). Next, a slow CO_2_ diffusion method was applied to grow the granular transition layer in the presence of Ca^2+^ and poly-(α,β)-DL-aspartic acid (PAsp) or poly(acrylic acid) (PAA). Subsequently, mineralization on the transition layer led to a prismatic-type overlayer. Such thin films bearing a three-layered structural heterogeneity show remarkably comparable mechanical properties to their biogenic counterparts. Furthermore, employment of a biomacromolecular soluble additive—silk fibroin (SF; from cocoons of the silkworm *Bombyx mori*) in the overgrowth procedure produced the exterior micro-/nano-texture, which reconciled brittleness and ductility in the prismatic-type mineral and generated under-water superoleophobicity. We therefore consider this general strategy to have a broad applicability in the fabrication of an enormous range of crystalline hybrid thin films with structural complexity and emerging functionalities.

### Prismatic–type CaCO_3_ thin films

**Fig. 2:**
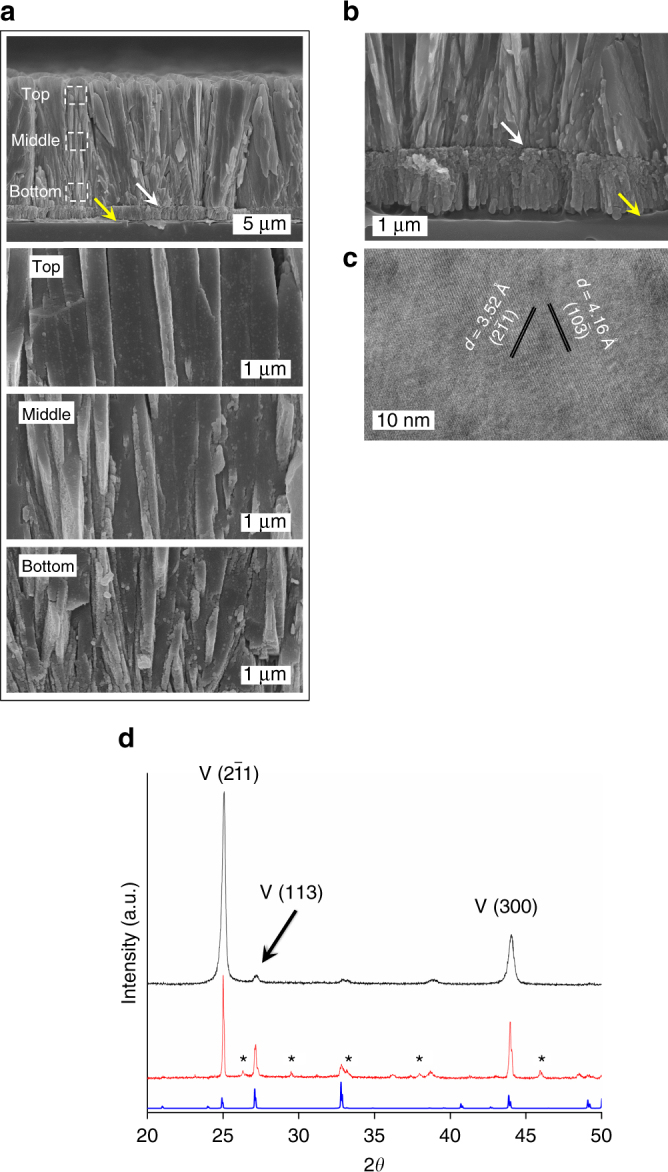
Characterization of the prismatic-type CaCO_3_ thin films. **a**, **b**, Cross-section SEM images showing the three-layered architecture of the thin film, composed of the PVA layer, the granular CaCO_3_-PAsp transition layer, and the prismatic CaCO_3_ overlayer in a bottom-to-top view. Three magnified cross-section SEM images of image **a** showing the gradual structural change in the overlayer in a top-to-bottom view. The white and yellow arrows in images **a**, **b** indicate the transition layer-overlayer junction and the transition layer-polymeric substrate interface, respectively. **c** High-resolution TEM image of a typical prism in the overlayer, which is assigned to vateritic CaCO_3_. **d** XRD patterns of prismatic CaCO_3_ (middle) & CaCO_3_-SF (upper) thin films and the vaterite standard (lower). Numerous aragonite/calcite peaks in the middle pattern were highlighted with asterisk marks. Indices in the high-resolution TEM **c** and the XRD pattern **d** were based on the *P*3_2_21 structure^28^, which led to the best fitting to the SAED pattern among several vaterite structures (Supplementary Fig. 13)

The cross-section scanning electron microscope (SEM) characterization of the product reveals a continuous, prismatic thin film with the uniform thickness, typically 10–20 μm, with the exact dimensions controlled by the mineralization period (Fig. 2a, b). Figure 2b reveals the presence of two clear interfaces within the three-layered architecture, namely the PVA matrix underneath, the granular transition layer, and the upper prismatic overlayer. A transmission electron microscope (TEM) image also discloses the clear interface between the intermediate granular transition layer and the upper prismatic overlayer (Supplementary Fig. 1; the sample was prepared by a focused ion beam treatment). It is noteworthy that the overlayer is composed of densely-packed microprisms with reinforcement orientation (Fig. 2a). A comparison of structural details at different positions along the vertical direction reveals that the overlayer shows the gradual structural change in a typical prismatic-type thin film. The initially-grown overlayer is about 100 nm in thickness, and adjacent prisms are slightly scattered instead of being perfectly vertically-oriented. With the height increase, the average diameters of the prisms increase to several hundreds of nanometers, while their number density decreases assumedly due to a coarsening process^24^. This gradual structural change gives rise to a uniform prismatic overlayer across the macroscopic distance. Both the slightly radial architecture and the gradual structural change disclose that the overgrowth passes through a competition growth pathway, as detected in numerous minerals and biominerals^25–27^. A top-view SEM image shows that the exterior surface of the overlayer is composed of closely-packed prisms, which are elongated columns in morphology (Supplementary Fig. 2b). Hence, SEM imaging confirms that the mesoscopic structural subunits of the overlayer are densely-packed prisms averagely aligning along the vertical direction of the thin film. The (high-resolution) TEM analysis confirms that each prism is single crystalline vateritic CaCO_3_, based on the hexagonal lattice of a density functional theory (DFT) structure^28^ (Fig. 2c and Supplementary Fig. 1b). The X-ray diffraction (XRD) pattern demonstrates dominance of vateritic CaCO_3_ in the overlayer with a preference for the (2$\bar 1$1) plane over others (Fig. 2d) with a percentage of 49.6 ± 1.3% (*N* = 5). Interference of other vateritic peaks can be attributed to the presence of cavities, as verified in microscopic images (Supplementary Fig. 2a, c, d). We note that vaterite—the least stable anhydrous CaCO_3_ polymorph—can exist in few biominerals^29,30^.

Preliminary experiments indicate that the annealing temperature of the PVA matrix (Supplementary Note 1 and Table 1), together with the use of PAsp or PAA in the fabrication of the transition layer, dictates both morphological and polymorphic outcomes of the transition layer and the overlayer (Supplementary Fig. 3a–f and 4a–f), in accordance with a previous study^31^. A continuous vateritic CaCO_3_-PAsp transition layer with the appearance of sporadic aragonitic domains was achieved when a PVA thin film annealed at 175 °C was employed as the substrate. Otherwise, the employment of other annealing temperatures nevertheless caused the decreased amount of vateritic polydomains (Supplementary Fig. 3a–d). The granular vaterite layer obtained was polycrystalline and spherulitic (Supplementary Fig. 4a–f). Hence, it is conceivable that the microenvironmental changes brought by the preadsorption of acidic polyelectrolytes on the partially hydrolyzed PVA matrix^32^ in the reacting mother liquor favor the nucleation of the vateritic transition layer and subsequently, have a crucial impact on its structural outcomes including the orientation and surface coverage^33^.

Comparison of the crystallographic information in the transition layer and that in the overlayer provides crucial evidence of the overgrowth process of the prismatic CaCO_3_ thin films. The XRD patterns evidence that the vateritic overlayer shows the similar orientational preference to the transition layer of the same polymorphic form (Fig. 2d and Supplementary Fig. 4c). It is therefore deducible that epitaxial match exists between the two layers, evidencing that the CaCO_3_-PAsp transition layer functions as the nucleation site for overgrowth. The clear interface between the two layers however, can be attributed to the morphological difference caused by the discrepant mineralization details, i.e., the involvement of polymer additives or not. For instance, the granular nature of the transition layer was due to mineralization in the presence of polyelectrolytes, while the overlayer characteristic of the oriented, single crystalline prisms was obtained in the absence of polymer additives.

It is remarkable that mineralization on a transition layer could lead to a continuous overlayer with the distinct mesostructural form. A comparison experiment showed that a mineralization process occurring on a PVA substrate led to spherulitic vateritic polycrystals (Supplementary Fig. 5a, b), shedding light on the indispensable role of the transition layer in obtaining an oriented, prismatic overlayer across the macroscopic distance. This result is in accordance with previous ones, where mineralization on semicrystalline polymers caused discontinuous mesostructural forms assumedly due to the limited driving force of heterogeneous nucleation^23,34,35^ and the related inhomogeneous feeding from the reacting solution phase^36^. On the other hand, other biomimetic studies verified that mineralization in the presence of the high concentration level of polymer additives facilitated the achievement of continuous granular layers comprised of both mineralized and polymeric constituents^37,38^. These thin films nevertheless lacked either distinct mesostructural forms or orientational reinforcement. In short, the granular transition layer, instead of functioning as a passive substrate, plays an active role in seeding the overgrowth of continuous prismatic CaCO_3_ thin films.

The granular transition layer in the current study is composed of binary constituents, namely minerals and polyelectrolytes. As a comparison, traditional seeds for fabrication of columnar thin films are composed of one-component and are the amorphous, solvated, or nanocrystalline form of the constituents in the overlayer^18,19,25^. Hence, the role of the polyelectrolytes in the transition layer needs clarification. After removal of the PAA constituent, a transition layer was employed for growth of the overlayer (Supplementary Fig. 6a, b). Nevertheless, instead of obtaining a continuous prismatic overlayer, sporadic vateritic polydomains were grown on the transition layer (Supplementary Fig. 6c, d). This result indicates that in the granular CaCO_3_-PAA transition layer, the PAA constituent increases the nucleation number density in the overgrowth procedure effectively, resulting in a continuous prismatic overlayer, while the mineralized constituent–vateritic CaCO_3_ offers structural succession to the overlayer of the same polymorphic form. Moreover, it is noteworthy to mention that the PAA constituent in the transition layer has an indirect impact on the orientational preference in the overlayer, as the increased number density of prisms in the overlayer facilitates the competition growth pathway^25^. The orientational relationship between the transition layer and the overlayer will be discussed in detail in this manuscript.

Vateritic, prismatic thin films were obtained in the current study. As a comparison, synthetic vateritic CaCO_3_ precipitated from the solution phase grew into hexagonal polycrystals composed of lamellar structural units. For instance, plate-shaped vateritic superstructures composed of hexagonal nanoplatelets were obtained in the presence of polymer additives, where the (001) facet of vateritic CaCO_3_ was exposed^39^. This distinct morphological difference from previous studies can be attributed to the template effect provided by the PVA substrate. For instance, Supplementary Fig. 5a, b shows that spherulitic vateritic polycrystals composed of radially-aligned prisms were precipitated on a PVA substrate. The seeded mineralization due to the presence of nucleus sites and the competition growth pathway, promoted the growth of a vertically-oriented, prismatic overlayer across the macroscopic distance (Supplementary Fig. 2a–c). Both vateritic superstructures employ prisms as structural units, though they are spatially different in alignments.

### Prismatic–type CaCO_3_–SF thin films

**Fig. 3:**
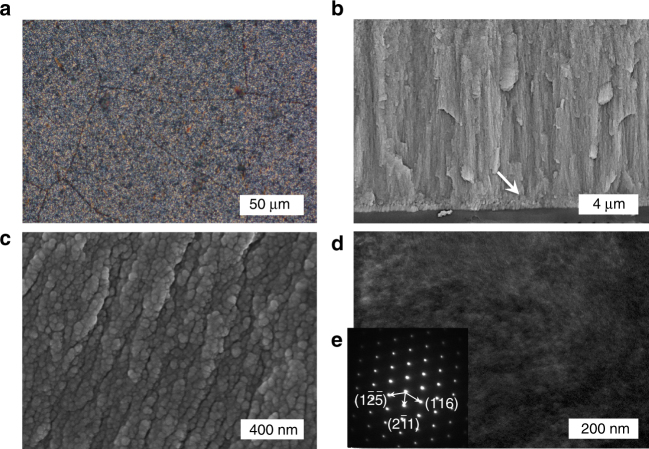
Characterization of the prismatic-type vateritic CaCO_3_-SF thin films. **a** POM image showing the uniform prismatic thin films over an area of hundreds of square micrometers in domain size. **b** Cross-section SEM image showing the structural information of the prismatic overlayer, where the boundary between the transition layer and the overlayer is highlighted by a white arrow. **c** Cross-section SEM image showing the granular nature of the overlayer. **d**, **e** TEM **d** and SAED **e** images of the prismatic overlayer. Indices in the SAED image **e** were based on the *P*3_2_21 structure^28^. The overgrowth occurred in the presence of 6 g L^−1^ SF

The presence of SF in the overgrowth procedure could lead to a prismatic-type layer carrying numerous structural changes. No SF gelation was detected both in the CaCO_3_-SF thin film and in the reacting mother liquor, revealing monomeric SF with small amounts of oligomers (Supplementary Fig. 7a–d). Hence, it functioned as a soluble additive in the overgrowth process. The CaCO_3_-SF overlayer is continuous and uniform across an area of at least hundreds of square micrometers in size (Fig. 3a); conversely, numerous cavities exist in the overlayer achieved in the absence of SF (Supplementary Fig. 2c, d). The prismatic overlayer, composed of nanograins high in packing density, is clearly observed (Fig. 3b, c). The different locations in the same microdomain of an overlayer exhibit the same growth orientation on the vertical direction of the (2$\bar 1$1) plane, as shown by spots in a selected area electron diffraction (SAED) pattern (Fig. 3d, e). This single crystalline character evidences that nanocrystalline structural subunits have the same global crystallographic orientation, indicating the mesocrystalline nature of the microdomain^40^. We note that the granular nature observed in our synthetic overlayers, due to the presence of a biomacromolecular soluble additive and dopant–SF, is characteristic of prismatic-type biominerals found in mollusk shells due to the presence of glycoproteins, though biogenic forms usually show a texture close to single crystals because of the low organic content^41^. The XRD pattern confirms that the CaCO_3_-SF overlayer shows the enhanced orientational preference of the (2$\bar 1$1) plane with a percentage of 77.4 ± 1.0% (*N* = 5) across the macroscopic distance, compared with its CaCO_3_ counterpart achieved in the absence of SF (Fig. 2d). The enhancement can also be attributed to the uniformity and continuity of the CaCO_3_-SF overlayer.

Characterization of a growing CaCO_3_-SF overlayer gives rise to the critical mechanistic understanding of the enhancement of orientational preference due to the presence of SF. A growing CaCO_3_-SF overlayer in the initial stage is characteristic of isolated surface nuclei assumedly due to the presence of the high supersaturation (Supplementary Fig. 8a). Hence, a mineralization period less than 8 h in the current study is inadequate for the formation of continuous overlayers. The morphology of the overlayer evolves with time, and microscopically, pre-existing nuclei and newly formed nuclei grow into isolated islands, which then spread and coalesce to a continuous thin layer (Supplementary Fig. 8b). It hence verifies that a long mineralization period is favorable for improving the spatial continuity of the overlayer. The same growth pathway was also observed in the overgrowth of the prismatic overlayer in the absence of SF. Nevertheless, the islands obtained in the absence of SF are relatively large in size and low in nuclei number density when compared with those obtained in the presence of SF (Supplementary Fig. 8c, d). This group of comparison experiments verifies that the presence of SF in the overgrowth procedure increases the nucleation rate effectively, leading to a continuous overlayer with decreased structural defects and increased orientational preference. The promotion of the epitaxial nucleation rate is assumedly due to the pre-adsorption of SF on the transition layer^42–44^.

Furthermore, the organic and inorganic constituents in the CaCO_3_-SF thin film were studied separately. The porous SF framework could be preserved by etching the inorganic component with a diluted hydrochloric acid solution (Supplementary Fig. 9a, b), while, a heating/calcination step was employed to remove SF in the overlayer to produce porous CaCO_3_ frameworks (Supplementary Fig. 9c, d). Both results confirm that SF is intimately incorporated in the prismatic overlayer. In addition, the synthetic vateritic CaCO_3_ thin film herein showed good thermal stability, as it could withstand a temperature at 400 °C for at least 24 h without transforming to other anhydrous CaCO_3_ polymorphic forms such as aragonitic and calcitic ones, as evidenced by the XRD pattern collected after the heating treatment (Supplementary Fig. 9e).

### Evidence of the competition growth pathway

**Fig. 4:**
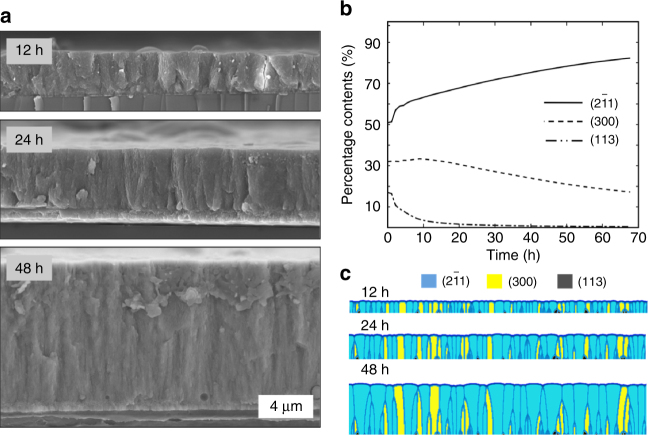
Growth mechanism of the prismatic-type CaCO_3_-SF thin films. **a** Three SEM images show growing overlayers different in thickness. **b** Phase-field simulation of a growing overlayer depicting the temporal evolutions of the film morphology with crystal anisotropy of (2$\bar 1$1), (300), and (113) planes. **c** Three snapshots showing the contents of (2$\bar 1$1), (300), and (113) planes against the mineralization time, where the three planes are illustrated with the light blue, yellow, and dark gray colors, respectively

Time-resolved XRD patterns of the CaCO_3_-SF overlayers indicate that the intensity ratio of the (2$\bar 1$1) and (300) peaks increases with the thickness increase (Table 1 and Fig. 4a), which again discloses that there exists a competition between multiple growth directions as previously found in biominerals^45,46^. The results of phase-field simulation validate that the synergetic effect of the seed selection in the granular layer and the competition among favorable growth directions lead to the oriented prismatic morphology (Fig. 4b, c). The competition growth model has been used extensively in the deposition of semiconductor thin films^18,25^, minerals^26,27^, and the prismatic-type biominerals found in avian eggshells^45,47^. Herein, this model was used to design biomimetic CaCO_3_ thin films, leading to oriented, uniform, prismatic overlayers in mimicking their biogenic counterparts found in mollusk shells.

**Table 1:** Relationship between area ratios of the (211) and (300) peaks in the time-resolved XRD patterns and the overlayer thickness

**Mineralization Period (h)**	**Overlayer thickness (μm)**	**Peak ratio**	**s.d**.
0	0	1.71	0.05
12	3.4	1.99	0.03
24	8.2	2.52	0.02
48	11.0	3.44	0.22

Data were collected in the prismatic overlayer achieved in the presence of 6 g L^−1^ SF. Five measurements were performed in different areas of the same sample to obtain average values

### Mechanical properties

**Fig. 5:**
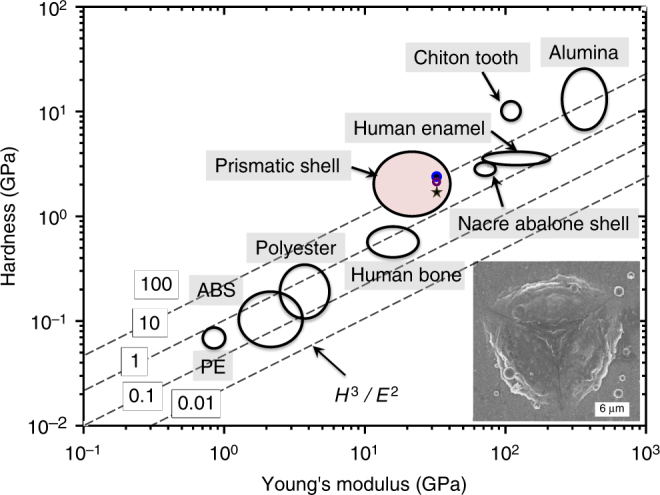
Ashby plot of the synthetic prismatic CaCO_3_ thin films and other materials. Blue spot: vateritic CaCO_3_ thin film; purple circle: CaCO_3_-SF thin film ([SF] = 4 g L^−1^); gray star: CaCO_3_-SF thin film ([SF] = 6 g L^−1^). The inserted image showing an exemplary residual indent of the Berkovich diamond tip in the CaCO_3_-SF overlayer

Remarkably, nanoindentation measurements (Supplementary Note 2) were employed to measure the hardness (*H*) and Young’s modulus (*E*) values of the synthetic, vateritic CaCO_3_ and CaCO_3_-SF overlayers. Both values were determined by analyzing load-displacement curves, where the exemplary ones of the CaCO_3_ and CaCO_3_-SF (i.e., [SF] = 6 g L^−1^) thin films are shown in Supplementary Fig. 10. For instance, the synthetic prismatic CaCO_3_ overlayer obtained in the absence of SF exhibits *H* and *E* values of 2.19 ± 0.2 and 32.5 ± 2.4 GPa (*N* = 18), respectively (Fig. 5). For comparison, the *H* and *E* values of the biogenic prismatic-type minerals found in mollusk shells are in ranges of 1–4 and 10–40 GPa, respectively^4^. Hence, the synthetic prismatic minerals studied herein exhibit comparable *H* and *E* values to biogenic minerals of the same type. The *H* and *E* values of the CaCO_3_-SF overlayer (i.e., [SF] = 4 g L^−1^) are 2.09 ± 0.10 and 33.9 ± 2.8 GPa (*N* = 9), respectively; while both values of the CaCO_3_-SF overlayer (i.e., [SF] = 6 g L^−1^) are 1.72 ± 0.21 and 33.4 ± 3.0 GPa (*N* = 15), respectively, which indicate a slightly decreased hardness for the highest SF concentration used (Fig. 5). In Fig. 5, it can be seen that while the hardness (2 GPa) of the prismatic layer is equal to that of the nacreous layer (2 GPa), the elastic modulus (33 GPa) is only about 50% of that for the nacreous layer (70 GPa), indicating that the nacreous layer is much stiffer. This finding is in qualitative agreement with other materials like silicon, where the tensile strength for a columnar polysilicon (1.31 GPa^48^ and 1.48–1.76 GPa^49^) was only found to be ca. 50% of that for the laminated polysilicon (2.44 GPa^48^ and 2.80–2.83 GPa^49^). However, these values should not be analyzed in an isolated way, since both nacreous and prismatic layers contribute to the outstanding mechanical properties of seashells due to the 90° angle between the anisotropic building units of both layers^10^.

Both biomimetic thin films show comparable *H* and *E* values with vateritic^50^ and calcitic CaCO_3_^51,52^ (For vateritic CaCO_3_ minerals, *H* = 0.4–2.8 GPa, *E* = 16–48 GPa; *H* values of calcite is in the same range, while its *E* value is ~ 80 GPa) due to the high reinforcement orientation between densely-packed crystalline prisms^4,9^. It is significant that the *H* values of the CaCO_3_-SF overlayers were largely maintained, though SF itself has much deceased hardness compared with the mineralized constituents. This can be convincingly attributed to the strong structural correlations between adjacent nanocrystalline structural subunits high in packing density, supported by XRD patterns, cross-sectional SEM images, and the SAED pattern (Figs. 2d and 3c–e). We foresee that both *H* and *E* values of biomimetic CaCO_3_ thin films can be enhanced further by optimizing microstructural and polymorphic information, as biomimetic calcitic CaCO_3_-hyperbranched polyglycidol hybrid thin films^53^ show the increased *H* value compared to their geologic counterparts^51,52^. Like its biogenic counterparts found in mollusk shells, the synthetic prismatic overlayers with orientational reinforcement can function as a protective shield from piercing force^41^.

It is assumed that the occlusion of a high amount of the SF constituents in the CaCO_3_-SF overlayer could theoretically increase the fracture toughness of the mineralized overlayer, as SF itself is a prevalent example of biopolymers with significant fracture toughness^54^. The thermal gravimetric analysis confirms that the total organic content in the CaCO_3_-SF thin film is as high as 12.9 wt% (i.e., [SF] = 6 g L^−1^). This value is close to the percentage of the SF occlusion, as the prismatic overlayer takes the vast majority of the total mass of the three-layered architecture. As a comparison, previous mechanical studies indicate that the prismatic-type biominerals composed of single crystalline columns are brittle in nature^41,55^, which can be attributed to the low percentage of the organic constituents and the specific mesostructural form. For instance, the organic constituents in the prismatic-type biominerals in the mollusk *Atrina rigida* are usually lower than 1 wt%^55^. Biogenic minerals, unlike their nacreous counterparts exhibiting remarkable toughness, serve as a protective shield from predaceous attacks from a sharp-clawed crustacean^4^.

The toughness of the vateritic CaCO_3_-SF overlayer (i.e., [SF] = 6 g L^−1^) characteristic of being flat on the nanoscale was next studied by nanoindentation. The pile-ups by indentation lead to plastic deformation on the exterior surface of the overlayer, and no microscopic crack or crack propagation is visible around the indent at high loads (>500 mN) (Fig. 5, inserted image). It is noteworthy that the granular nature of the highly-crystalline CaCO_3_-SF overlayer (Fig. 3c) results in excellent fracture strength, which can tolerate the presence of flaws in the thin coating^56^. The presence of the SF constituents between adjacent nanograins dissipates indentation energy efficiently and hence, prevents localized nanocracks from further propagation^57,58^. The same crack prohibition mechanism has been observed in biogenic and synthetic nacre^14,41^ and micelle-occluded calcitic crystals^59^. As a comparison, cracks start to appear and propagate on the exterior surface of a prismatic-type calcitic biomineral – the mollusk *Atrina rigida* ^55^ at high loads. It is noteworthy that in addition to the occlusion of SF constituents, SF can toughen the mineralized thin film by varying the single crystalline prisms with respect to the prismatic micro-/nano-texture, the latter of which largely dissipates the strain because of the presence of the interfaces between adjacent nanograins^56^. Therefore, the occlusion of the SF constituents reconciles brittleness and ductility in the synthetic prismatic overlayer.

### Under-water superoleophobicity

**Fig. 6:**
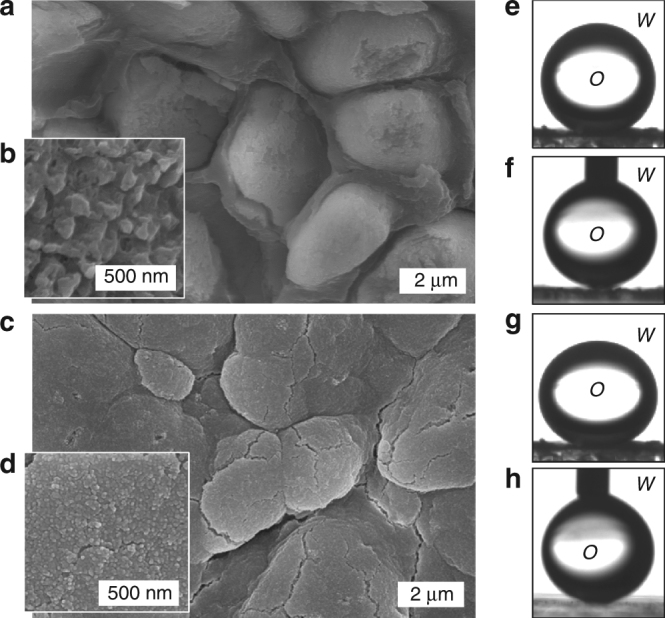
Comparison of exterior texture and wettability of *Pinctada margaritifera* & CaCO_3_-SF thin film. **a**–**d** Top-view SEM images of the exterior micro-/nano-textures surfaces of *Pinctada margaritifera* **a**,** b** & a prismatic CaCO_3_-SF thin film (i.e., [SF] = 4 g L^−1^) **c**, **d**. **e**–**h** Photographs showing the superoleophobicity and low adhesion of the corresponding *Pinctada margaritifera* **e**, **f** and a prismatic CaCO_3_-SF thin film **g**, **h**. Abbreviations *W* and *O* represent water and 1,2-dichloroethane, respectively. Each oil droplet is 3 μL in volume

Biominerals are usually multifunctional materials with emerging properties, many of which are only slowly discovered^1^. The under-water superoleophobicity has been found in numerous aquatic organisms including fish scales and clam shells, which bear a micro-/nano-texture in the hydrophilic mineralized exterior layer^60^. Herein, the prismatic-type layer of *Pinctada margaritifera* shows under-water superoleophobicity with a contact angle of 160.1 ± 3.8° (*N* = 5) (Fig. 6e, f). We reveal that the synthetic, prismatic CaCO_3_-SF thin films (i.e., [SF] = 2 and 4 g L^−1^) bear comparable values of 152.8 ± 1.3° and 158.2 ± 0.8° (*N* = 5), respectively (Fig. 6g, h). The under-water superoleophobicity becomes more excellent with the increase of the [SF]. This function of biogenic and synthetic thin films can be attributed to the micro-/nano-texture found in the highly mineralized exterior layer hydrophilic in nature (Fig. 6a–d). However, when continuously improving the SF content, the contact angle of the prismatic CaCO_3_-SF thin film (i.e., [SF] = 6 g L^−1^) decreased to 142.4 ± 0.6° (*N* = 5). The failure of achieving the under-water superoleophobicity is likely due to its smoothness on the nanoscale. In short, the morphology and structural changes caused by doping of SF (Supplementary Fig. 11) have a big influence on the under-water superoleophobic property of the prismatic CaCO_3_-SF thin film. On the other hand, the contact angle of the prismatic CaCO_3_ layer is 147.8 ± 1.1° (*N* = 5) in the same measurement due to the absence of nanotextures (Supplementary Fig. 12), and for a silicon surface it is 132.7 ± 5.5° (*N* = 5). The biomimetic superoleophobic interface with low affinity to oil drops can find applications in areas such as oil-water separation, anti-fouling, and anti-scaling.

## Discussion

In summary, we successfully fabricate oriented, prismatic-type CaCO_3_ layers continuous on a macroscopic scale by using a total morphosynthetic approach. This mineralization approach highlights the crucial role of the transition layer in generation of continuous, prismatic overlayers. Moreover, the competition growth pathway that leads to numerous biogenic prismatic-type minerals is evidenced in the synthetic thin films of the same type with reinforcement orientation. Such prismatic-type thin films bearing a three-layered spatial structural heterogeneity are extremely difficult to fabricate via alternative assembly and top-down approaches. This universal biomimetic approach to significant mechanical and wetting properties can find potential applications in fabrication of advanced coating and biomedical materials.

## Methods

### Polymer coating

Poly(vinyl alcohol) (PVA, the average *M*_*w*_ = 1.5–1.9 × 10^5^ g mol^−1^, 87–89% hydrolyzed, Sigma-Aldrich) was used as the substrate for CaCO_3_ mineralization. PVA thin films were prepared as follows. A volume of 20 μL PVA-DMSO (AR, Sinopharm Chemical Reagent) solution (1 wt%) was dropped onto the hydrophilic cover glass (1.2 × 1.2 cm^2^, pretreated with Piranha solution) and spread uniformly, followed by vacuum drying to evaporate the solvent, to yield the PVA thin film. The thin films were used as prepared or annealed at different temperatures for 1 h before use (see results of PVA characterization in Supplementary Table 1).

### Deposition of the transition layer

In a typical procedure to achieve the transition layer, the mineralization was conducted by the slow diffusion of CO_2_ (g) (based on the slow decomposition of NH_4_HCO_3_ (s, AR, Sinopharm Chemical Reagent) in a volume of 4 mL 20 mM CaCl_2_ (aq) (AR, Sinopharm Chemical Reagent) in the presence of 1.0 × 10^−3^ wt% poly-(α,β)-DL-aspartic acid sodium salt (PAsp, average *M*_*w*_ = 2.0–11.0 × 10^3^ g mol^−1^, Sigma-Aldrich) or poly(acrylic acid sodium salt) (PAA, partial sodium salt solution, average *M*_*w*_ = 2.1 × 10^3^ g mol^−1^, 61–65 wt% in water, Sigma-Aldrich) as the additive in a closed desiccator for 24 h, based on a previous study^22^. A PVA thin film was positioned at the bottom of the reacting mother liquor for mineralization of the transition layer. Subsequently, the transition layer, together with the underneath PVA thin film and the glass substrate, was removed and rinsed twice with degassed purified water (obtained from Millipore, Direct-Q3 and boiled for 0.5 h to remove CO_2_ before use) before being used for characterization or overgrowth.

### Overgrowth

For the overgrowth of the prismatic, vateritic layer, the solution was prepared using CaCl_2_ or CaCl_2_–silk fibroin (SF, from cocoons of the silkworm *Bombyx mori*) solutions, where the concentration of SF varied between 1–6 g L^−1^. Purification of SF was based on the standard procedure provided by Kaplan and coworkers^61^. The concentration of Ca^2+^ was constant at 20 mM for all overgrowth experiments. A transition layer was positioned at the bottom of the reacting mother liquor for mineralization of the overlayer.

### Other related experiments

The resulting prismatic-type hybrid thin film was heated on a heating block at 400 °C in open air to remove any organic ingredient. Treatment of the product with 1 mM HCl (aq) was used to remove the mineralized ingredients. A CaCO_3_-PAA transition layer was immersed in methanol for 5 h to remove the PAA constituents. Subsequently, the substrate was rinsed with ethanol for three times. Finally, the substrate was employed for the overgrowth tests.

### Characterization

An Olympus BX53 optical microscope equipped with polarizers was used for the OM & POM observation. SEM images were collected using a Hitachi SU-70 electron microscope. Samples were prepared by breaking the thin film into pieces, and then sticking them onto double-side conducting adhesive tape. Samples were coated with a thin layer of gold using a sputter coater for 30 s (*i.e*. for SEM acquisition at 15 kV). Focused Ion Beam (FIB)-TEM sample preparation: We used a Zeiss Neon 40EsB to prepare the TEM samples. To protect the surface during milling and lift-out a bar of SiO_2_ was deposited on the sample using gas-assisted deposition. After lift-out and transfer onto the TEM grid the sample was polished to the final thickness using a 50 pA current. X-ray diffraction patterns were collected using an X’pert PRO, PANalytical, X-ray diffractometer with Cu-K_α_ radiation. Diffraction patterns were generated under machine operation at 40 mA and 40 kV and using a step size at 0.016°, and calculation of peak ratios was based on the peak height. Raman spectra were recorded using a Labram HR Evolution system (Horiba) equipped with a 532 nm laser. The Laser power and beam size were ~0.25 mW and 1 μm, respectively. The polymorph assignment of CaCO_3_ was verified according to reference^62^. For underwater contact angle measurements, surfaces were measured at ambient temperature on a DSA100 contact angle/interface system (DataPhysics) using the sessile drop method. After the surface was immersed in water, a 3 μL 1,2-dichloroethane droplet was dropped carefully on the surface for the contact angle measurement. The oil droplet was slowly lifted to show its adhesion with the surface. The average value of three measurements performed at different positions of the same sample was adopted as the underwater contact angle. The ATR FT-IR spectra from 4000 to 500 cm^−1^ were collected on a Nicolet iS10 FT-IR spectrometer (Thermo Scientific). Circular dichroism experiments were performed on a JASCO J-810 spectropolarimeter (the reacting mother liquor was diluted until the SF concentration was ~ 0.1 g L^−1^ to obtain a good signal). The IR and CD spectroscopy results were analyzed according to reference^63^. Analytical ultracentrifugation was performed with a XL-I ultracentrifuge (Beckman–Coulter) at 38,000 rpm and 25 °C using 12 mm Ti double sector centerpieces (Nanolytics). Rayleigh interference optics and UV–vis absorption optics were applied simultaneously. The interference data were used for the evaluation of the sedimentation coefficient distribution using the software SEDFIT (http://www.analyticalultracentrifugation.com/default.htm).

### Mechanical properties

A Nano Indentation (Agilent G200, US) with a Berkovich diamond probe was utilized to perform the nanoindentation tests on the prepared specimens. To ensure that the indentations were performed on calcium carbonate spherules, the indentation locations were selected manually under an optical microscope at a magnification factor of 1000. With a theoretical displacement resolution of 0.01 nm, the indentation tests were performed using the continuous stiffness measurement (CSM) technique with an indentation depth of 500 nm. During the loading process, the strain rate was controlled at a constant 0.5 nm s^−1^ with a loading resolution of 50 nN.

### Phase-field simulation

For simulation of the oriented overgrowth of the prismatic, vateritic CaCO_3_ thin film, a phase-field model was carried out by considering the interfacial free energy anisotropy of different growth orientations^64,65^. A number of 80 seeds with (2$\bar 1$1), (300), or (113) planes were randomly positioned on the substrate. Their number percentage, based on the peak ratio in the XRD patterns (Table 1 in the manuscript), is 51%, 32%, and 17%, respectively. See details in Fig. 4b, c in the manuscript and the Supplementary Movie 1.

### Data availability

The data that support the findings of this study are available from the corresponding author upon reasonable request.

## Electronic supplementary material

Supplementary Information

Description of Additional Supplementary Files

Supplementary Movie 1
