# Supplementary material for: Total morphosynthesis of biomimetic prismatic-type CaCO3 thin films
Source: Nat Commun. 2017 Nov 9;8:1398. doi: 10.1038/s41467-017-01719-6 (PMC5680295; doi:10.1038/s41467-017-01719-6)
Supplement: Supplementary file 1 — Supplementary Information [file 41467_2017_1719_MOESM1_ESM.pdf]

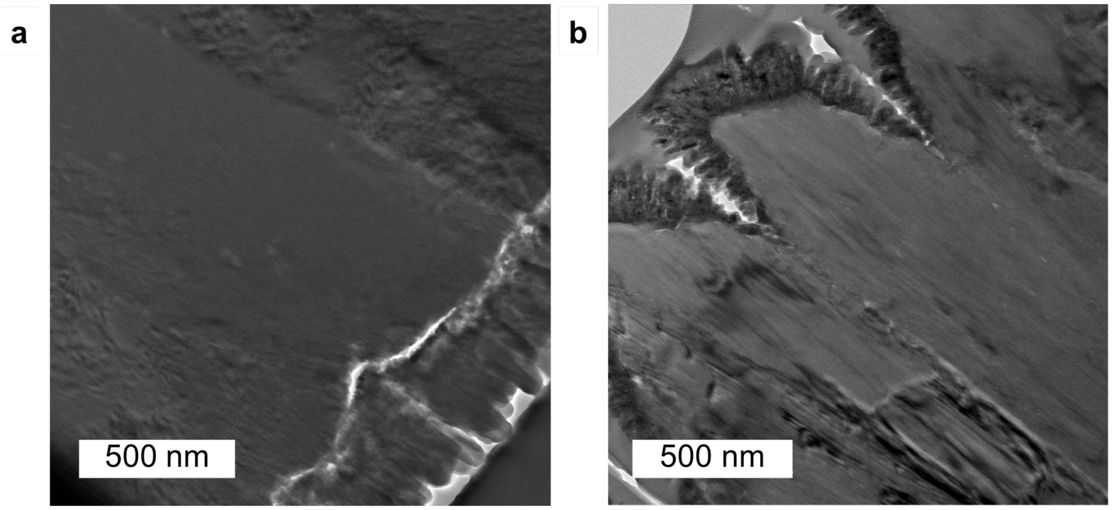

Supplementary Figure 1. Transmission electron microscopy (TEM) images of prismatic-type vateritic $CaCO_3$ overlayers. **a**, Interface between the granular $CaCO_3$-PAsp (PAsp denotes poly-(α,β)-DL-aspartic acid sodium salt) transition layer and the prismatic-type $CaCO_3$ overlayer. **b**, Zoom-in image of the prismatic-type overlayer. A focused ion beam technique was applied for fabrication of the sample.

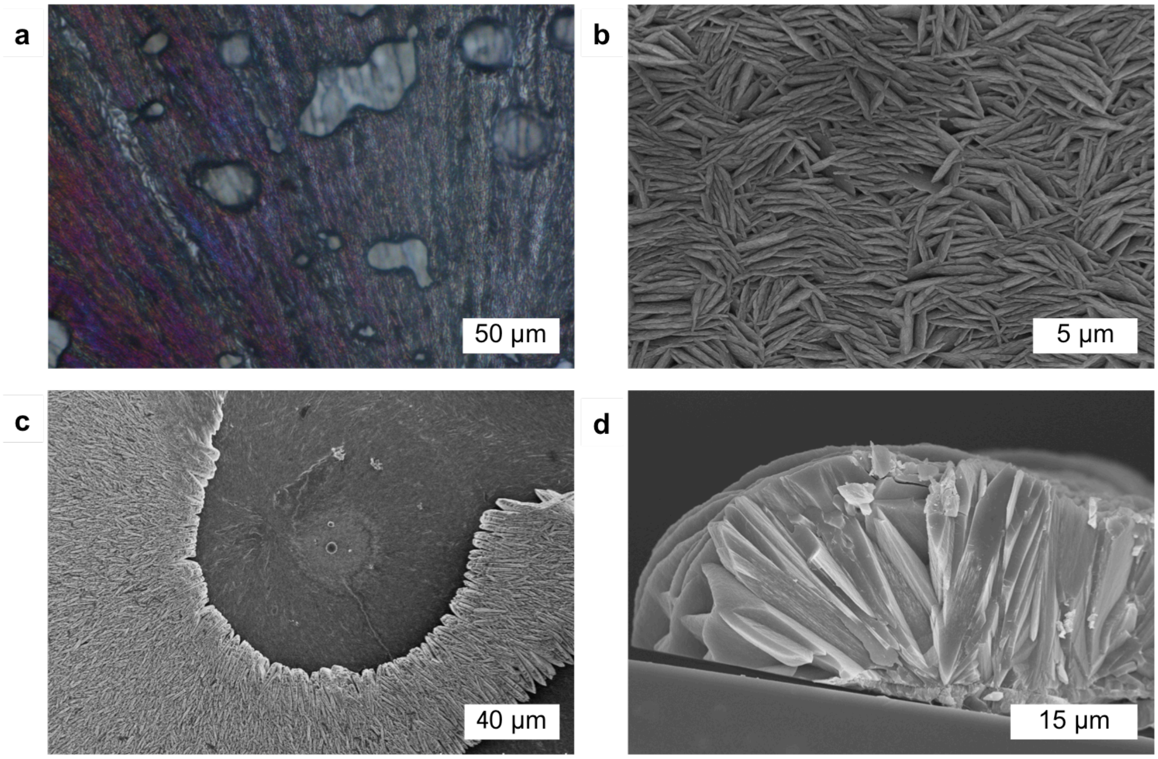

Supplementary Figure 2. a-c, Top-view polarized optical microscopy (POM) (**a**) and scanning electron microscopy (SEM) images (**b**-**d**) of the exterior surfaces of the prismatic-type, vateritic $CaCO_3$ thin film fabricated in the absence of silk fibroin (SF). **c**-**d**, Top- (**c**) and side-view SEM (**d**) images showing the existence of cavities of the prismatic-type vateritic $CaCO_3$ thin films.

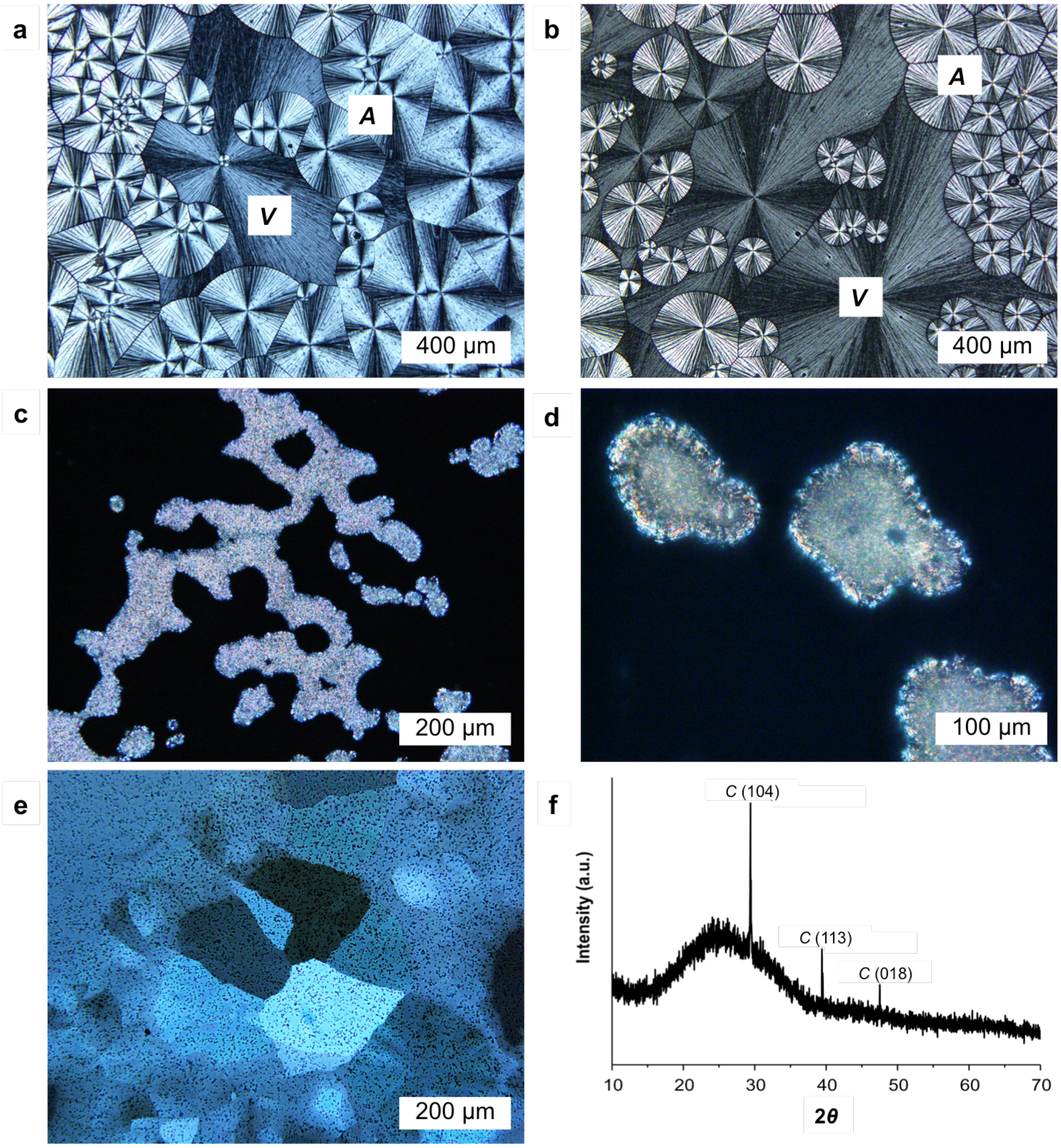

Supplementary Figure 3. Relationship between the poly(vinyl alcohol) (PVA) substrates and the structural outcome of the mineralized layers. a-d, Annealing temperature effect of the PVA substrate to the polymorphic outcomes of the transition layers (**a-b**) and overlayers (**c-d**). **a-b**, POM images of the $CaCO_3$-PAA (PAA denotes poly(acrylic acid sodium salt)) transition layers by using PVA substrates annealed at 155°C (**a**) and 165°C (**b**) for 1h. The abbreviations "*V*" and "*A*" in both images denote vateritic and aragonitic $CaCO_3$, respectively. Vateritic and aragonitic $CaCO_3$ domains were confirmed by Raman microscopy[1]. **c-d**, POM images of the overlayers fabricated using PVA substrates annealed at 185°C (**c**) and 190°C (**d**) for 1h (The transition layers used in the current study were composed of the $CaCO_3$-PAA hybrid). SF was absent in fabrication of the prismatic-type overlayers (**c** & **d**). **e-f**, Characterization of the polycrystalline $CaCO_3$-PAA hybrid thin film grown on the glass substrate. Both POM image (**e**) & X-ray diffraction (XRD) pattern (**f**) show the polycrystalline thin film is calcite. The abbreviation "*C*" in image **f** represents calcite.

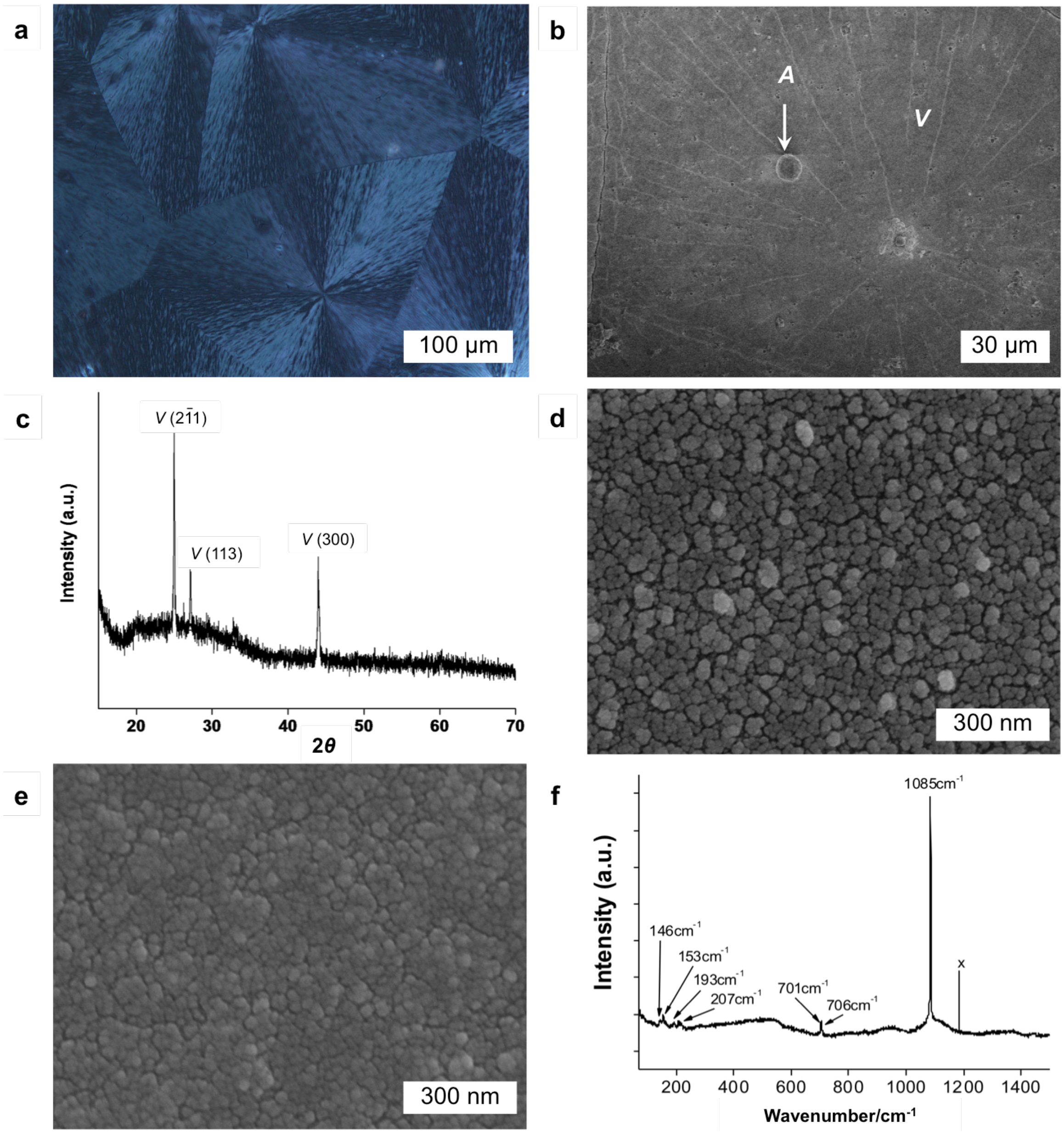

Supplementary Figure 4. Characterization of the granular transition layer fabricated using PAA or PAsp as the soluble additive. a-b, Overview POM (**a**) and SEM (**b**) images show the dominance of the vateritic $CaCO_3$-PAsp spherulitic domain with scattered aragonitic spherulites. The abbreviations "*V*" and "*A*" in image **b** denote vateritic and aragonitic $CaCO_3$, respectively. **c**, XRD pattern indicates that the vateritic $CaCO_3$-PAsp granular transition layer is partially crystalline vateritic $CaCO_3$. The abbreviation "*V*" in image **c** represents vaterite. **d-e**, High magnification SEM images show the granular nature of vateritic (**d**) and aragonitic (**e**) $CaCO_3$ domains, respectively. **f**, Raman spectrum of the aragonitic $CaCO_3$ domain.

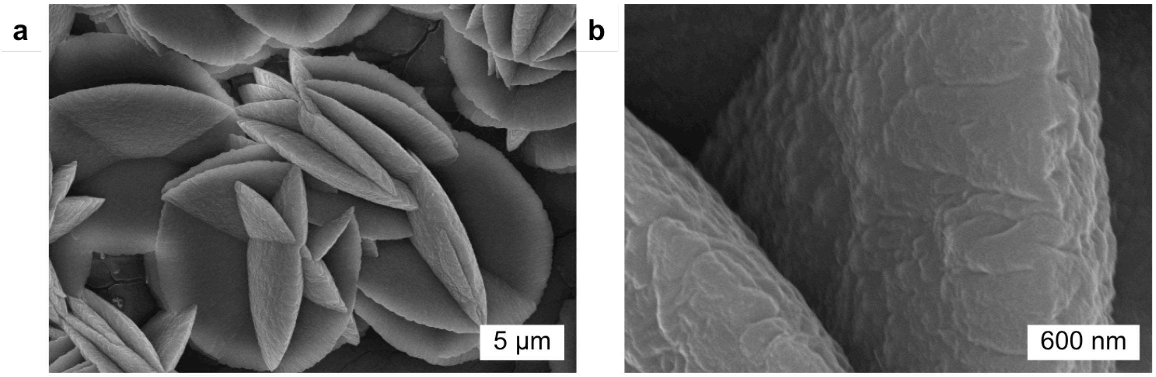

Supplementary Figure 5. Overview (**a**) and structural details (**b**) of spherulitic vateritic $CaCO_3$ crystals deposited on the PVA substrate.

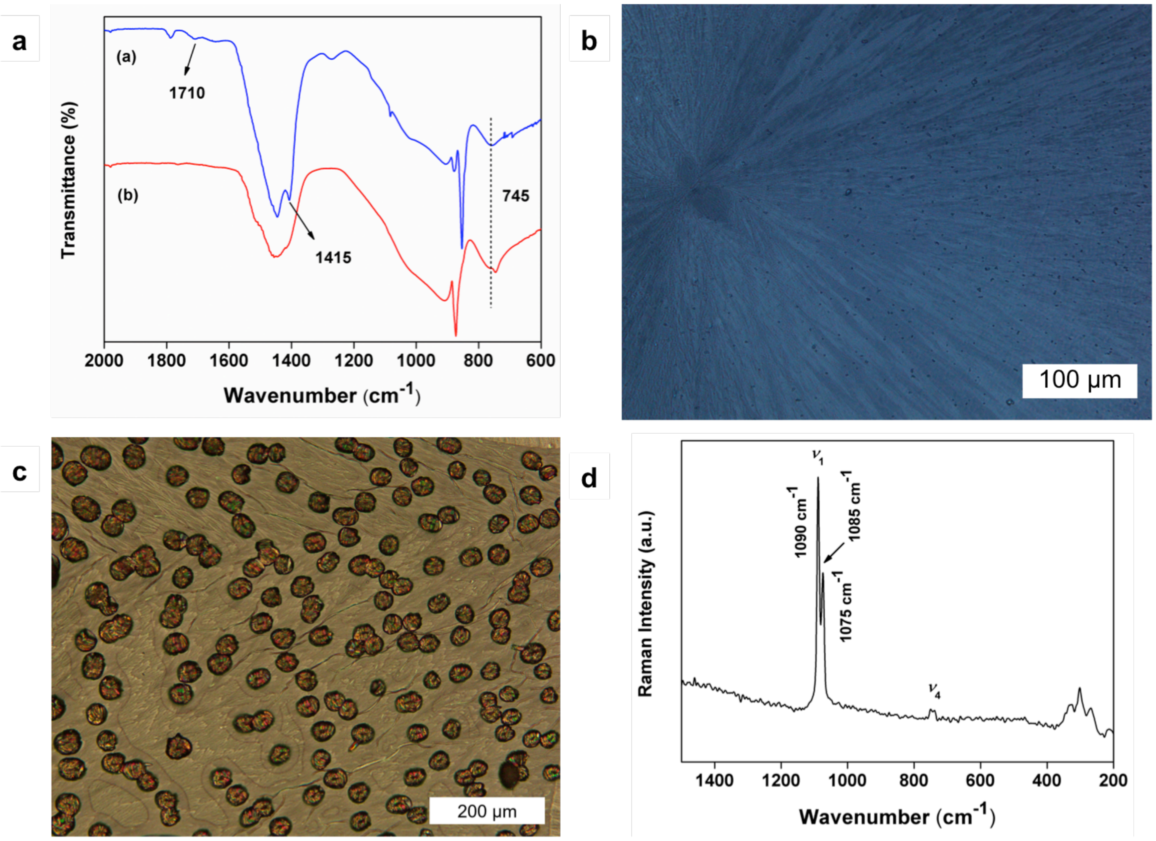

Supplementary Figure 6. Characterization of the transition layer before and after PAA removal & overgrowth on the transition layer after PAA removal. a, FT-IR spectra showing the chemical information of the $CaCO_3$-PAA transition layer before (blue) and after (red) PAA removal by the methanol treatment. **b**, POM image indicating the granular nature of the transition layer after PAA removal. **c-d**, POM image (**c**) and Raman spectroscopy (**d**) showing the structural character and vateritic nature of the overlayer grown on the transition layer after PAA removal.

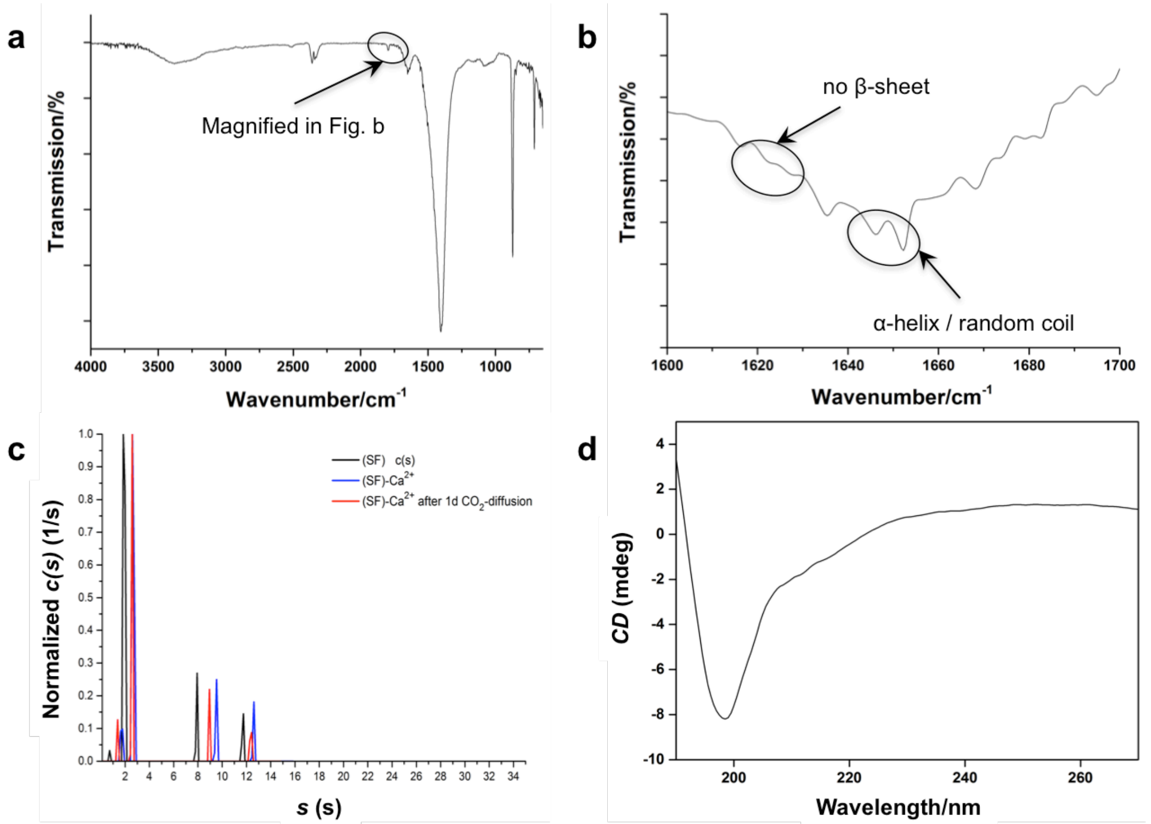

Supplementary Figure 7. Detection of SF form after mineralization. a-**b**, FT-IR spectrum showing no β-sheet formation in the prismatic-type $CaCO_3$-SF thin film. **c**-**d**, Sedimentation coefficient distribution from Analytical ultracentrifugation (AUC) (**c**) and the circular dichroism spectrum[2] (**d**) of the liquor collected before and after mineralization show no gelation signal of SF. AUC (**c**) reveals mainly monomeric SF with a few higher oligomers (6 g L^{-1}). Also after addition of Ca^{2+} and in the mother liquor after mineralization, SF stays mainly monomeric and the sedimentation coefficient distributions of SF in presence of Ca^{2+} and in the mother liquor after mineralization overlap. Both distributions are just shifted to higher sedimentation coefficients with respect to SF by density increase due to the complexation of Ca^{2+} to SF. The $CaCl_2$-SF aqueous solution in mineralization contains 6 g L^{-1} SF and 20 mM $CaCl_2$.

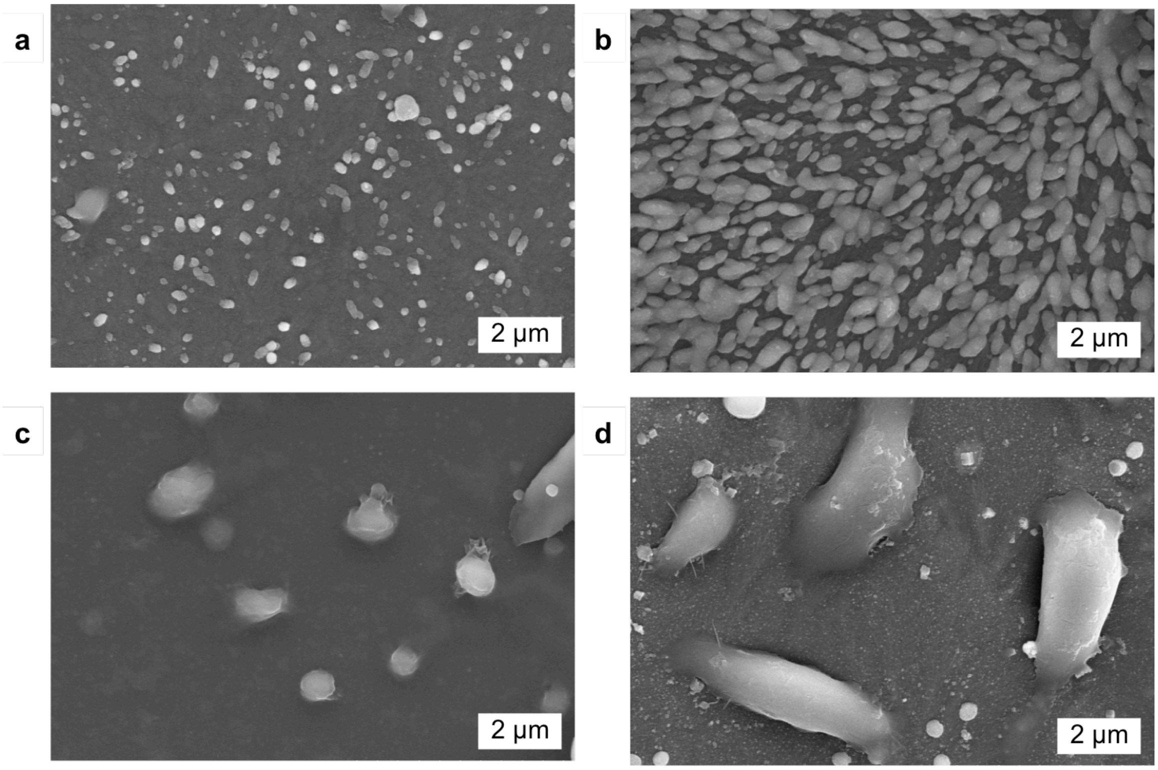

Supplementary Figure 8. Time-resolved studies of the overgrowth of prismatic-type overlayers. **a**-**b**, SEM images show typical structural information of the $CaCO_3$-SF overlayer at 6 h (**a**) and 8 h (**b**), respectively. The $CaCl_2$-SF aqueous solution in mineralization contained 6 g L^{-1} SF and 20 mM $CaCl_2$. **c**-**d**, SEM images show typical structural information of the vateritic $CaCO_3$ overlayer in the absence of SF at 6 h (**c**) and 8 h (**d**), respectively.

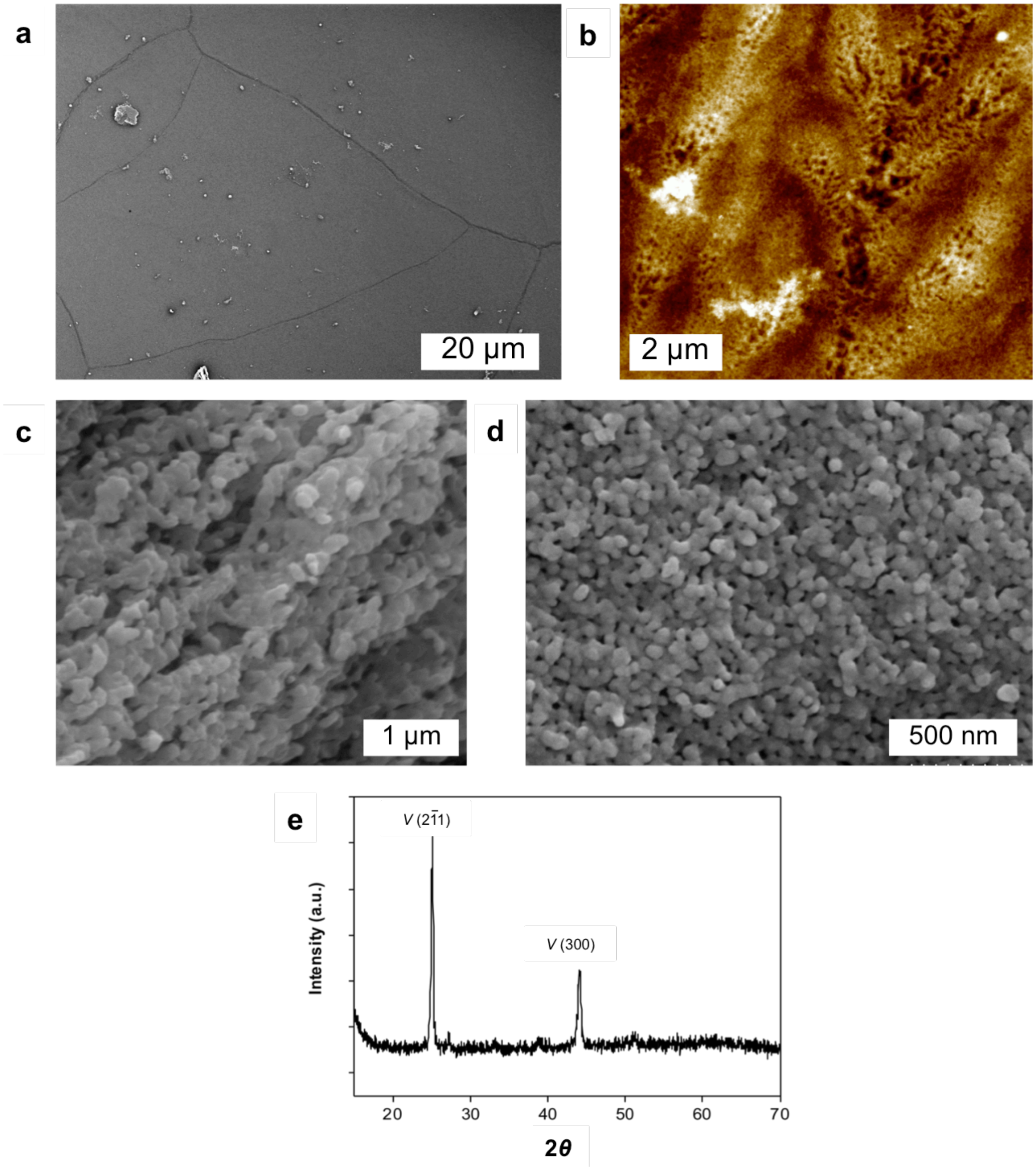

Supplementary Figure 9. Characterization of porous SF & mineralized frameworks. **a**-**b**, SEM (**a**) & atomic force microscopy (AFM) (**b**) images of the porous SF film after the dissolution of the mineralized ingredients. **c**-**d**, Cross-sectional (**c**) and top-view (**d**) SEM images show a porous and fragile prismatic-type thin film after an annealing process at 400°C in the open air to remove the soft ingredients. **e**, XRD pattern of the prismatic-type thin film after SF removal, in which the abbreviation "*V*" denotes vaterite. Data were collected from the thin films fabricated in the presence of 6 g L^{-1} SF.

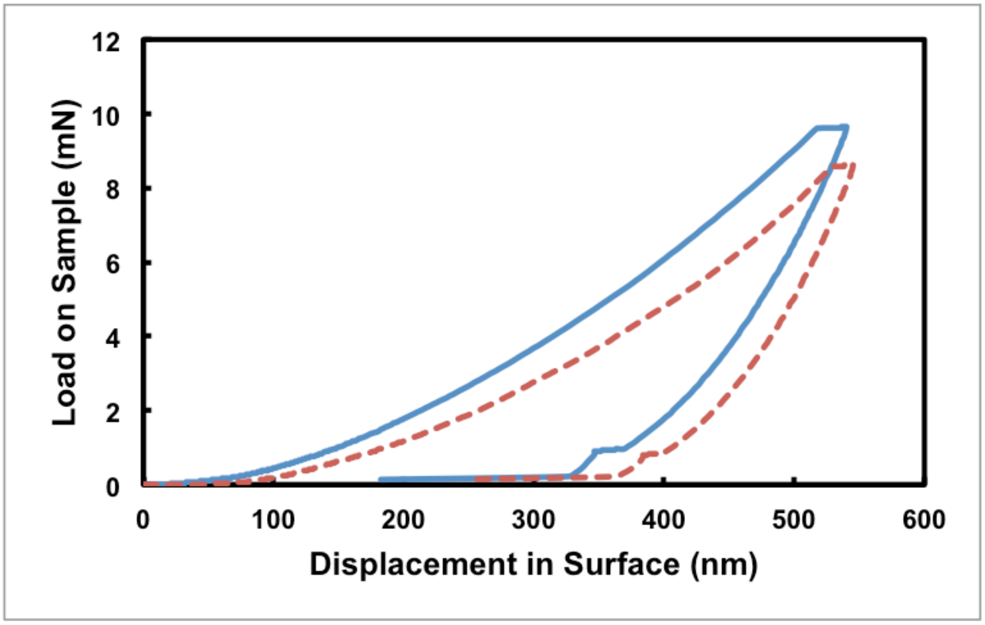

Supplementary Figure 10. Exemplary nanoindentation curves of the prismatic-type thin films. The solid and dotted curves were obtained in the absence of SF and in the presence of 6 g L^{-1} SF, respectively.

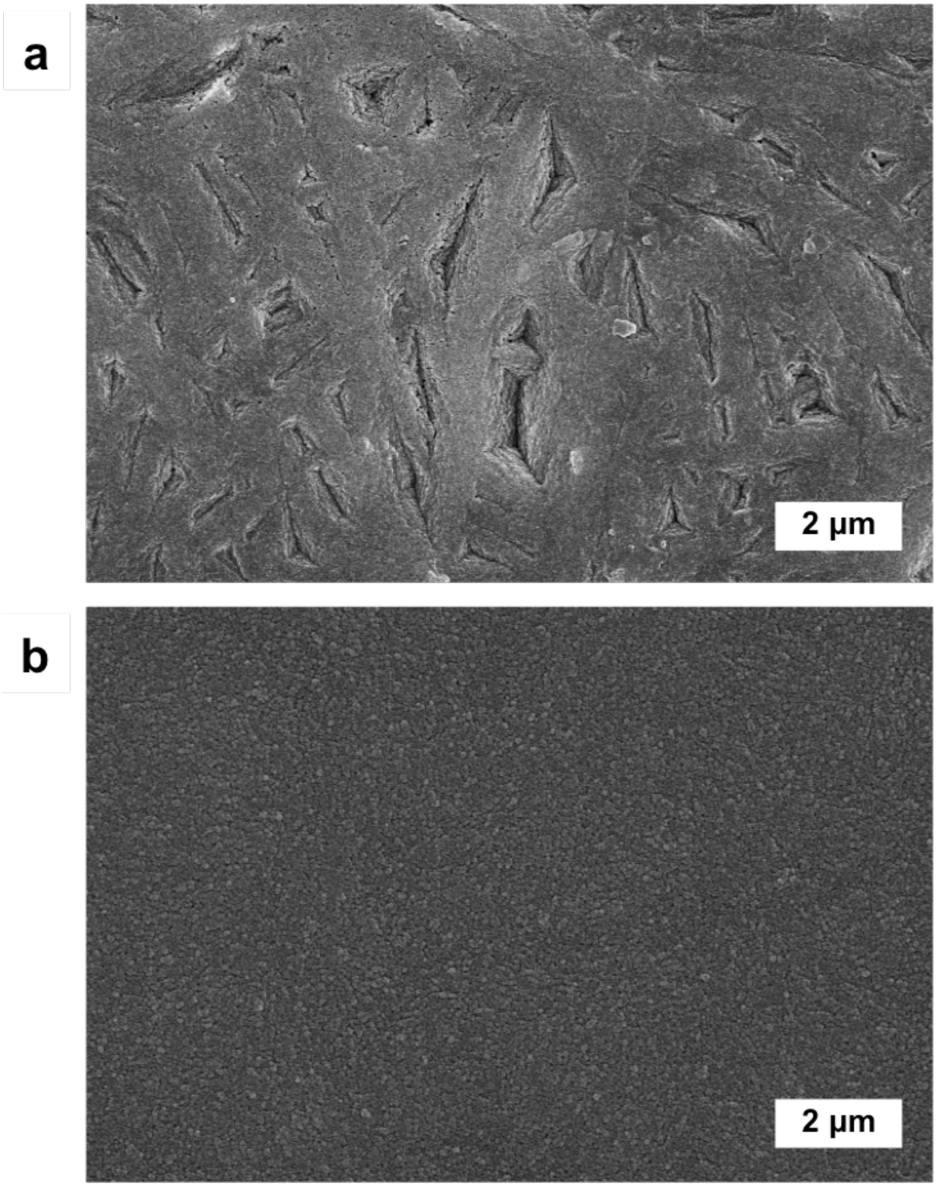

Supplementary Figure 11. SF concentration effect on the exterior texture of the prismatic-type overlayers. Top-view SEM images of the columnar films achieved in the presence of SF with the [SF] at 2 g L^{-1} (**a**) and 6 g L^{-1} (**b**).

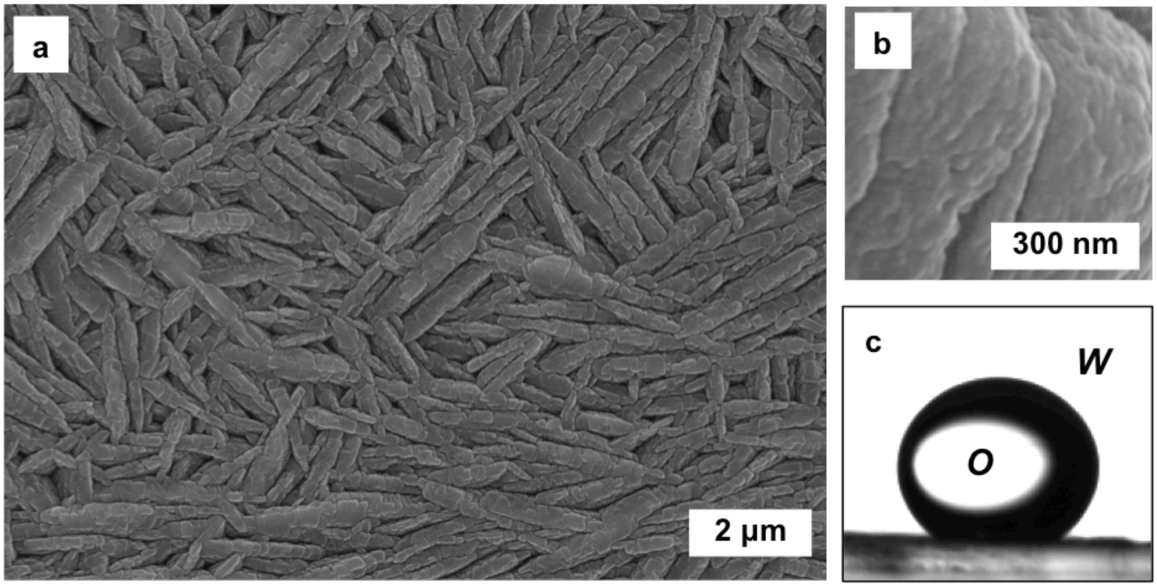

Supplementary Figure 12. Relationship between the exterior textures of the prismatic, vateritic $CaCO_3$ thin film and its under-water superoleophobicity. a-b, Top-view SEM images of the exterior micro-textured surfaces of the prismatic-type $CaCO_3$ thin film. **c**, Photograph showing an under-water oil droplet on the $CaCO_3$ thin film. Abbreviations "*W*" and "*O*" represent water and 1,2-dichloroethane, respectively. Each oil droplet is 3 μL in volume.

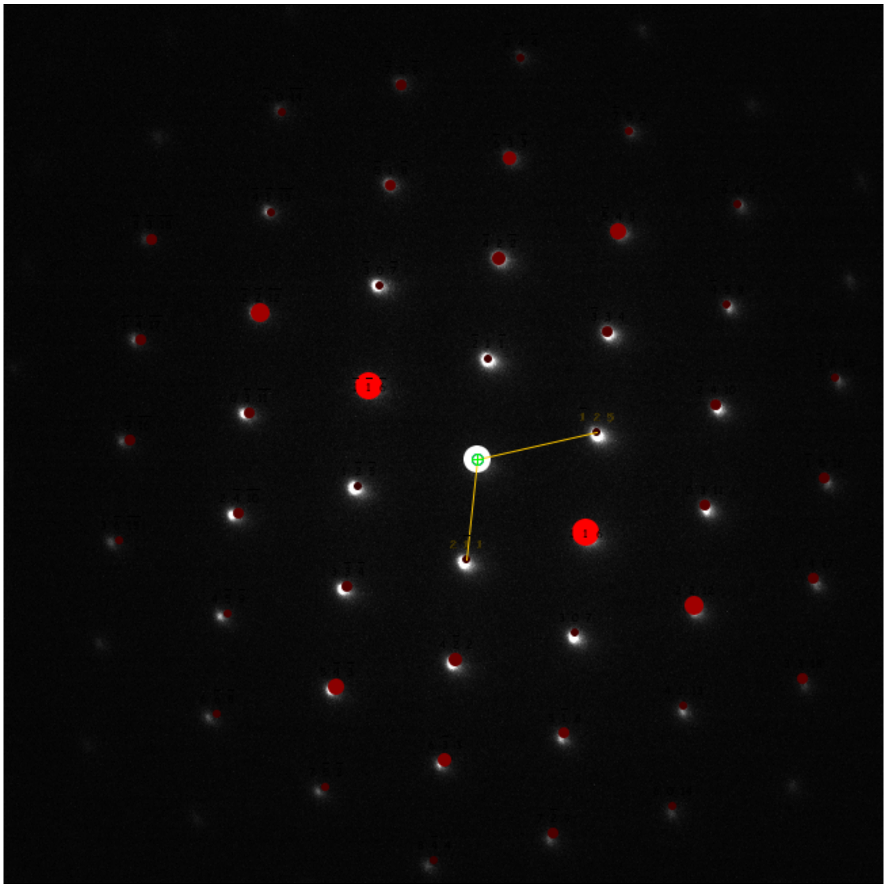

Supplementary Figure 13. Selected area electron diffraction solutions for the vaterite structure based on the Demichelis model ($P3_221$)[3]. Other models[3-7] only led to partial fitting.

Supplementary Note 1

Comments on the annealing temperature effect of the PVA substrate on the structrual outcome of the mineralized layers. It is conceivable that the microenvironmental changes, which are brought in by the preadsorption of acidic polyelectrolytes on the partially hydrolyzed PVA matrix[8] in the reacting mother liquor, have a crucial impact on the structural outcome of the transition layer and the overlayer[9]. As mentioned in the manuscript, a continuous vateritic transition layer was produced when a PVA thin film annealed at 175°C was applied for the mineralization. Higher annealing temperatures caused discontinuous prismatic, vateritic domains (Supplementary Fig. 3c-d). This incompleteness can be partially attributed to the high crystallinity of PVA (Supplementary Table 1), which causes water resistance and deteriorates the wetting behaviour of the reacting mother liquor during the formation of the transition layer[10]. By comparison, an annealing temperature at 170°C or lower caused the coexistence of the vateritic and aragonitic domains in the transition layer (Supplementary Fig. 3a-b). Hence, the annealing temperature was found to have a profound influence on the complex interplay between the hydrated PVA matrix and reacting mother liquor in the presence of the polyelectrolyte selected during the formation of the transition layer.

Supplementary Table 1. Characterization of the PVA substrates showing annealing temperature plotted against contact angle & crystallinity.

Annealing Temp. (°C)	Contact angle (°)	s.d. (%)	Crystallinity (%)*	s.d. (%)
as prepared	56.3	0.5	26.6	4.7
160	50.8	1.06	43.0	1.1
175	46.6	1.08	49.9	0.9
190	42.6	1.51	56.7	1.2

At least three parallel tests were performed in each experiment to achieve the average values.
*The crystallinity of PVA thin films was measured through the analyses of their infrared spectra[10].

Supplementary Note 2

Analysis of mechanical properties

The hardness H and reduced Young's modulus E_r for the samples investigated can be directly determined based on analysis of load displacement data using the following equations[11]:

$$H = F_m / A_c \quad (1)$$

$$1/E_r = 2\beta\sqrt{A_c/\pi}/S \quad (2)$$

$$1/E_r = (1-v_i^2)/E_i + (1-v_s^2)/E_s \quad (3)$$

where F_m is the force at maximum load, A_c is the contact area (A_c = 24.56 h^2 for a perfect tip, with h denoting the indentation depth), β is a correction factor depending on the tip geometry (β = 1.034 for the Berkovich indenter tip), S is the contact stiffness, E_i and E_s are the Young's modulus of the indenter and the sample respectively, and ν_i and ν_s denote Poisson ratios for the indenter and the sample respectively. E_i = 1141 GPa and v_i = 0.07 were used for the diamond indenter tips and a value of v_s = 0.3 was assumed for calcium carbonate crystals[12]. Each of the two specimens was subjected to nine indentation tests, with abnormal variations excluded prior to calculating the average values of the test results.

References[13-20] were used for the Ashby plot in Figure 5 in the article.

Supplementary References

1 Wehrmeister, U., Soldati, A. L., Jacob, D. E., Häger, T. & Hofmeister, W. Raman spectroscopy of synthetic, geological and biological vaterite: a Raman spectroscopic study. *J. Raman Spectrosc.* **41**, 193-201 (2010).

2 Matsumoto, A. *et al.* Mechanisms of silk fibroin sol-gel transitions. *J. Phys. Chem. B* **110**, 21630-21638 (2006).

3 Demichelis, R., Raiteri, P., Gale, J. D. & Dovesi, R. A new structural model for disorder in vaterite from first-principles calculations. *CrystEngComm* **14**, 44-47 (2012).

4 Meyer, H. J. Uber Vaterit und seine Struktur. *Angew. Chem.* **1959**, 678-679 (1959).

5 Kamhi, S. On the structure of vaterite $CaCO_3$. *Acta Crystallogr.* **16**, 770-772 (1963).

6 Wang, J. & Becker, U. Structure and carbonate orientation of vaterite ($CaCO_3$). *Am. Mineral.* **94**, 380-386 (2009).

7 Mugnaioli, E. *et al.* Ab Initio Structure Determination of Vaterite by Automated Electron Diffraction. *Angew. Chem. Int. Ed.* **51**, 7041-7045 (2012).

8 Hodge, R. M., Edward, G. H. & Simon, G. P. Water absorption and states of water in semicrystalline poly(vinyl alcohol) films. *Polymer* **37**, 1371-1376 (1996).

9 Addadi, L., Moradian-Oldak, J. & Weiner, S. Molecule-Crystal Recognition in Biomineralization: Studies Using Synthetic Polycarboxylate Analogs. *ACS Symp. Ser.* **444**, 13-27 (1991).

10 Kenney, J. F. & Willcockson, G. W. Structure–Property relationships of poly(vinyl alcohol). III. Relationships between stereo-regularity, crystallinity, and water resistance in poly(vinyl alcohol). *J. Polym. Sci., Part A: Polym. Chem.* **4**, 679-698 (1966).

11 Oliver, W. C. & Pharr, G. M. An improved technique for determining hardness and elastic modulus using load and displacement sensing indentation experiments. *J. Mater. Res.* **7**, 1564-1583 (1992).

12 Ramsay, D. B., Dickinson, G. H., Orihuela, B., Rittschof, D. & Wahl, K. J. Base plate mechanics of the barnacle Balanus amphitrite (=Amphibalanus amphitrite). *Biofouling* **24**, 109-118 (2008).

13 Taylor, J. D. & Layman, M. The mechanical properties of bivalve (Mollusca) shell structures. *Palaeontology* **15**, 73-87 (1972).

14 Currey, J. D. & Taylor, J. D. The mechanical behaviour of some molluscan hard tissues. *J. Zool.* **173**, 395-406 (1974).

15 Rho, J.-Y., Tsui, T. Y. & Pharr, G. M. Elastic properties of human cortical and trabecular lamellar bone measured by nanoindentation. *Biomaterials* **18**, 1325-1330 (1997).

16 Cuy, J. L., Mann, A. B., Livi, K. J., Teaford, M. F. & Weihs, T. P. Nanoindentation mapping of the mechanical properties of human molar tooth enamel. *Arch. Oral Biol.* **47**, 281-291 (2002).

17 Weaver, J. C. *et al.* Analysis of an ultra hard magnetic biomineral in chiton radular teeth. *Mater. Today* **13**, 42-52 (2010).

18 Malinova, K. *et al.* Production of $CaCO_3$/hyperbranched polyglycidol hybrid films using spray-coating technique. *J. Colloid Interface Sci.* **374**, 61-69 (2012).

19 Kunitake, M. E., Mangano, L. M., Peloquin, J. M., Baker, S. P. & Estroff, L. A. Evaluation of strengthening mechanisms in calcite single crystals from mollusk shells. *Acta Biomater.* **9**, 5353-5359 (2013).

20 Olson, I. C. et al. Crystal lattice tilting in prismatic calcite. *J. Struct. Biol.* **183**, 180-190 (2013).
