# Supplementary material for: Total morphosynthesis of biomimetic prismatic-type CaCO3 thin films
Source: Nat Commun. 2017 Nov 9;8:1398. doi: 10.1038/s41467-017-01719-6 (PMC5680295; doi:10.1038/s41467-017-01719-6)
Supplement: Supplementary file 2 — Description of Additional Supplementary Files [file 41467_2017_1719_MOESM2_ESM.pdf]

Description of Additional Supplementary Files

File Name: Supplementary Movie 1
Description: Depicts the change of the intensity ratios of the three peaks during overgrowth, which discloses that there exists a competition between multiple growth directions.
